# Association of Vertebral Artery Hypoplasia and Vertebrobasilar Cerebrovascular Accident

**DOI:** 10.3390/medicina58091189

**Published:** 2022-08-31

**Authors:** Augenijus Vilimas, Virginija Gaigalaitė, Mykolas Urbonas, Dalius Jatužis

**Affiliations:** 1 Faculty of Medicine, Vilnius University, LT-03101 Vilnius, Lithuania; 2 Center of Neurology, Faculty of Medicine, Vilnius University, LT-03101 Vilnius, Lithuania

**Keywords:** vertebral artery hypoplasia, posterior circulation infarction, ultrasound

## Abstract

*Background and Objectives*: Vertebral artery hypoplasia (VAH) is a controversial risk factor for cerebral infarction. The aim of this study was to analyze the prevalence of vertebral artery hypoplasia and to evaluate its association with vertebrobasilar cerebrovascular accidents. *Materials and Methods*: The study was conducted in the Neurology Departments of the Republican Vilnius University Hospital from 2015 to 2020. Data of 742 subjects (133 patients with posterior circulation infarction or vertebral artery syndrome (PCI/VAS), 80 patients with anterior circulation infarction (ACI) and 529 control subjects with no symptoms of cerebrovascular accident) were analyzed. Ultrasound examination of the extracranial internal carotid and vertebral arteries (VA) was performed, risk factors were recorded. *Results*: The mean age of the subjects was 64.51 ± 13.02 years. In subjects with PCI/VAS the diameter of VA was smaller, and the prevalence of VAH was higher compared to those in subjects with ACI and in the control group. A higher degree of VAH in subjects younger than 65 years of age increased the risk of PCI/VAS. Subjects with non-dominant VA diameter of 2.7–2.9 mm had 2.21 times higher risk of PCI/VAS, subjects with non-dominant VA diameter of 2.5–2.6 mm had 2.36 times higher risk of PCI/VAS, and subjects with non-dominant VA diameter of 2.2–2.4 mm had 4.12 times higher risk of PCI/VAS compared with subjects with non-dominant VA diameter of ≥3 mm. Among patients with PCI/VAS those with VAH had lower rates of ischemic heart disease compared with patients with normal VA diameter. There was no difference in the rates of other risk factors between PCI/VAS patients with and without VAH. *Conclusions*: Vertebral artery hypoplasia is not a rare finding in individuals without symptoms of cerebrovascular accident, but more frequent in patients with vertebrobasilar cerebral infarction or vertebrobasilar artery syndrome. Vertebral artery hypoplasia can be considered a risk factor for posterior circulation infarction in subjects under 65 years of age.

## 1. Introduction

Cerebral infarction is one of the most acute social, economic and health care problems in Lithuania. The etiology of ~30% of cerebral infarctions remains unknown [1,2]. Advancements in diagnostics and identification of new risk factors for cerebral infarction could improve the prevention of cerebral infarction and reduce the morbidity and mortality from cerebral infarction. Ultrasound examination of cervical blood vessels (the vertebral and carotid arteries) is a non-invasive procedure that can be routinely performed and help detect hemodynamically significant arterial changes, useful in disease course prediction and monitoring response to treatment.

Vertebral artery hypoplasia is a controversial risk factor for cerebral infarction: discussions continue regarding what vertebral artery diameter can be considered hypoplastic and affecting cerebral blood flow, and whether vertebral artery hypoplasia is an independent risk factor for cerebral infarction. The pathogenetic mechanism of cerebral infarction in vertebral artery hypoplasia is also not completely understood.

The aim of this study was to analyze the prevalence of vertebral artery hypoplasia and to evaluate its association with vertebrobasilar cerebrovascular accident.

## 2. Materials and Methods

Subjects hospitalized in the Neurology Departments of the Republican Vilnius University Hospital from 2015 to 2020 and diagnosed with vertebrobasilar (brainstem) artery syndrome (ICD-10-AM G45.0) or cerebral infarction due to cerebral artery thrombosis (ICD-10-AM I63.3) were invited to participate in the study.

Inclusion criteria:

1. Adult subjects of both sexes;
2. Diagnosed with vertebrobasilar artery syndrome or cerebral infarction;
3. Agreed to participate in the investigation and signed an informed consent form.

Exclusion criteria:

1. Patients with intracerebral hemorrhage (ICD-10-AM I61);
2. Patients with recurrent cerebral infarction (ICD-10-AM I63);
3. Internal carotid artery stenosis > 50% or occlusion.

Individuals examined but not diagnosed with cerebrovascular accident were included into the control group.

Ultrasound examination of the extracranial internal carotid and vertebral arteries was performed with Aloka Prosound F75 diagnostic ultrasound system using a 7.5 MHz linear array transducer. The extracranial portion of VA was assessed along its entire length. VA diameter was measured in the V2 segment (foraminal) to avoid larger artifacts, possible in the V3 (extraspinal) and V1 (pre-foraminal) segments. Patients with cerebrovascular accident were examined by magnetic resonance imaging (MRI) and magnetic resonance angiography (MRA) using a 1.5 Tesla MRI (GE Optima MR450w 1.5T MRI System).

We evaluated different groups of VA diameters: less than 2.2 mm, 2.2–2.4 mm, 2.5–2.6 mm, and 2.7–2.9 mm and less than 3 mm.

Risk factors (arterial hypertension, diabetes, ischemic heart disease, atrial fibrillation) were recorded.

The study sample calculation was based on assumption that the prevalence of VAH among adults without neurological symptoms is 9.5% when VA diameter is measured by ultrasound, and the prevalence of VAH among adults with cerebral infarction is 19.1% [3,4]. The level of significance (probability of type I error) of 0.05, the power of 0.8 and the ratio of the control and case groups of 2.48 were selected. A sample calculation using the OpenEpi calculator showed that the study sample should include at least 531 subjects.

Data were analyzed using IBM SPSS Statistics (Statistical Package for Social Sciences) version 20 and Microsoft Excel. In the description of data frequency and relative frequency for categorical variables, mean and standard deviation, 95% of the confidence intervals (CI) for continuous variables are provided. The chi-square test was used to compare distribution of categorical variables between groups. One-way analysis of variance procedure (ANOVA) was used to compare means of three or more independent samples. Least significant difference (LSD) was used for multiple post hoc comparisons. Linear regression analysis was used to evaluate the effect of an independent variables (predictors) on the dependent variable. Logistic regression analysis was used to determine variables predicting the binary outcome. A significance level of 0.05 was chosen.

Vilnius Regional Biomedical Research Ethics Committee granted approval No. 158200-15-767-281 to conduct the study on 13 January 2015.

## 3. Results

A total of 1109 individuals were invited to participate in the study. A total of 367 individuals did not meet the inclusion criteria. Data of 742 subjects were analyzed. A total of 307 (41.4%) subjects were men and 435 (58.6%) women.

A total of 213 subjects experienced cerebrovascular accident: A total of 133 subjects had posterior circulation infarction or VAS (PCI/VAS) and 80 subjects had anterior circulation infarction (ACI). A total of 529 control subjects had no evidence of cerebrovascular accident.

The mean age of the subjects was 64.51 ± 13.02 years. Subjects with PCI/VAS were younger compared with subjects with ACI (*p* < 0.001) and with subjects without symptoms of cerebrovascular accident (*p* < 0.001) (Table 1). In subjects with PCI/VAS the right VA diameter, the left VA diameter, the mean total VA diameter, and the diameter of non-dominant VA were smaller comparing with those of subjects with ACI (*p* = 0.033, *p* = 0.007, *p* = 0.001 and *p* < 0.001, respectively) and with those of the control group (*p* = 0.003, *p* = 0.001, *p* < 0.001 and *p* < 0.001, respectively) (Table 1). The diameter of vertebral arteries did not differ in subjects with ACI and in the control group (Table 1).

The prevalence of non-dominant VA diameter of <3 mm was 56% among subjects without symptoms of cerebrovascular accident (Table 1).

The prevalence of non-dominant VA diameter of <3 mm, the prevalence of non-dominant VA diameter <2.7 mm and the prevalence of non-dominant VA diameter of <2.5 mm was statistically significantly higher in patients with PCI/VAS compared to the control group (*p* < 0.001, *p* < 0.001 and *p* < 0.001, respectively) and compared to patients with ACI (*p* = 0.003, *p* = 0.011 and *p* = 0.003, respectively) (Table 1). We did not find statistically significant differences in the prevalence of non-dominant VA diameter of <2.2 mm between patients with PCI/VAS and patients with ACI and control (Table 1). The prevalence of VAH did not differ between subjects with ACI and the control group.

**Figure 1:**
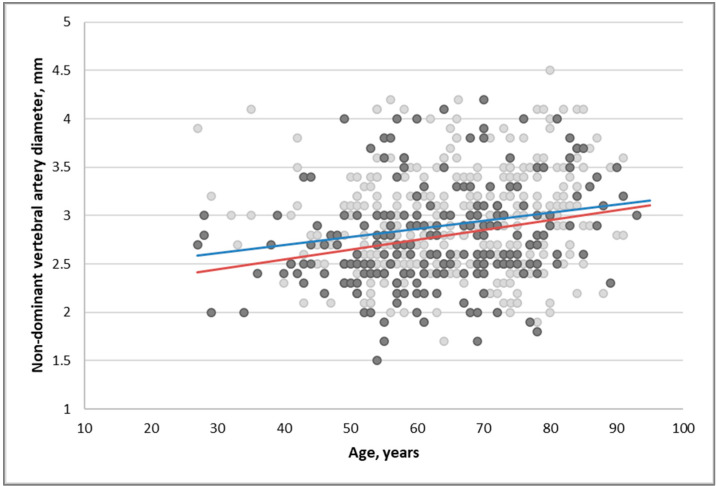
Association of non-dominant vertebral artery diameter and age in the group of patients with cerebral infarction or vertebrobasilar artery syndrome and subjects without symptoms of cerebrovascular accident. Red reference line represents patients with cerebral infarction or vertebrobasilar artery syndrome, blue reference line represents subjects without symptoms of cerebrovascular accident.

The diameter of non-dominant VA increased with age in the group of patients with cerebrovascular accident (β = 0.01 (95% CI 0.005–0.015), *p* < 0.001) and in the control group (β = 0.008 (95% CI 0.005–0.012), *p* < 0.001) (Figure 1).

Logistic regression revealed that wider non-dominant VA and older age are associated with lower risk of PCI/VAS (non-dominant VA diameter OR = 0.47; 95% CI: 0.31–0.70; *p* < 0.001 and age OR = 0.97; 95% CI: 0.96–0.99; *p* = 0.004). Further analysis demonstrated that a higher degree of VAH in subjects younger than 65 years increased the risk of PCI/VAS. Subjects with non-dominant VA diameter of 2.7–2.9 mm had 2.21 times higher risk of PCI/VAS compared with subjects with non-dominant VA of ≥3 mm. Subjects with non-dominant VA diameter of 2.5–2.6 mm had 2.36 times higher risk of PCI/VAS and subjects with non-dominant VA of 2.2–2.4 mm had 4.12 times higher risk of PCI/VAS compared with subjects with non-dominant VA ≥ 3 mm. In subjects ages 65 years and older no such association was found (Table 2).

A total of 177 (83.1%) patients with cerebrovascular accident had arterial hypertension, 31 (14.6%) had diabetes, 50 (23.5%) had ischemic heart disease and 27 (12.7%) had atrial fibrillation. Subjects 65+ years of age more frequently had hypertension (94.7% vs. 73.7%, *p* < 0.001), diabetes (20% vs. 10%, *p* = 0.043) ischemic heart disease (42.1% vs. 8.5%, *p* < 0.001) and atrial fibrillation (23.2% vs. 4.2%, *p* < 0.001) compared to those in subjects younger than 65 years.

The prevalence of diabetes, ischemic heart disease, and atrial fibrillation in patients with PCI/VAS was statistically significantly lower compared to patients with ACI (Table 3).

Among patients with PCI/VAS those with VA diameter of <3 mm had lower rates of ischemic heart disease compared with patients with normal VA diameter (11.1% and 29.4%, *p* = 0.012, respectively). There was no statistically significant difference in the rates of other risk factors between PCI/VAS patients having VAH and without VAH.

## 4. Discussion

In recent years multiple studies have pointed out an association between VAH and PCI [5,6], indicated that subjects with VAH have lower net VA flow volume and higher frequency of VA flow insufficiency [3,7,8]. VAH appears to be a pre-disposing factor for atherosclerotic stenosis in the posterior circulation [9]. Patients with PCI are at higher risk of early recurrent stroke, especially those with vertebrobasilar stenosis or hypoplasia [10].

In agreement with other studies our study revealed significantly higher prevalence of VAH in patients with PCI/VAS compared to that in patients with ACI and with subjects without evidence of cerebrovascular accident. A retrospective analysis of 529 patients with cerebral infarction by Park et al. showed that ipsilateral VAH is highly associated with PCI [11]. Perren et al. confirmed that among 725 first-ever stroke patients, those with PCI had VAH significantly more frequently compared to patients with infarctions in other location [12]. Similarly, Chi et al. noted significantly higher rate of VAH in patients with PCI than in patients with ACI [13].

VAH prevalence in patients with PCI and ACI detected in our study is compared with results of other studies in Table 4.

The prevalence of VAH in patients with PCI circulation infarction ranged from 13% to 45% [1,4,13,14,15,16]. In our study the rate of non-dominant VA diameter of <2.5 mm in patients with PCI/VAS was similar to that found by Kulyk et al., who studied patients of a similar age as we did (mean age of patients with PCI was 60 ± 13 years compared with 60.34 ± 12.77 years in our study) [4]. In the studies conducted by Perren et al., Sauer et al., the prevalence of VAH was lower than in our study, but the average age of the patients was also higher: 65.7 and 68 years, respectively [12,15]. Frequency of VA diameter of <3 mm among patients with PCI/VAS in our study was higher than in other, which could be explained by different definitions of VAH, differences of study population by age and race.

Discussions on whether VAH is an independent risk factor or aggravates the vascular risk for PCI, continue. We were not able to find information from any prospective study related to the relative risk of VAH in cerebral infarction development. The majority of studies were retrospective investigations of patients with cerebral infarction. Hu et al. investigated 841 patients with first-ever diagnosed cerebral infarction and demonstrated that VAH (VA diameter of <2 mm by computed tomography or MRA) is an independent risk factor for having PCI, increasing PCI odds ratio two times [14]. A retrospective analysis of 235 young patients with acute ischemic stroke by Yang et al. confirmed that VAH is an independent risk factor for PCI stroke in young patients, increasing risk of PCI 2.21 times [17].

The results of our study demonstrate that a higher degree of VAH was associated with an increased risk of PCI/VAS development: subjects with non-dominant VA diameter of 2.7–2.9 mm had 2.21 times higher risk of PCI/VAS, subjects with non-dominant VA diameter of 2.5–2.6 mm had 2.36 times higher risk of PCI/VAS and subjects with non-dominant VA diameter of 2.2–2.4 mm had 4.12 times higher risk of PCI/VAS compared to subjects with VA diameter of ≥3 mm, but such association was noticed only in the group of subjects younger than 65 years. This finding is likely to be associated with the fact that in younger patients with low impact of other risk factors the hypoperfusal circulatory disorder mechanism is the main risk factor of PCI/VAS. In older people, atherosclerosis related risk factors were the main contributors leading to circulatory disorders, rather than the decreased blood flow through VA. It is worth to mention that our investigation revealed that the diameter of non-dominant VA increased with age in groups of patients with cerebrovascular accident and in subjects without evidence of cerebrovascular accident. Positive correlation between age and the diameter of VA was reported by Chen et al. in a study of 1000 subjects and by Jeng et al. in 447 subjects [3,18].

We did not find any statistically significant differences in the rates of hypertension, diabetes, atrial fibrillation between PCI/VAS patients with and without VAH. Patients with PCI/VAS with VA diameter of <3 mm had lower rates of ischemic heart disease compared to patients with normal VA diameter (11.1% and 29.4%, *p* = 0.012, respectively). Szarazova et al. did not find correlation between conventional vascular risk factors (hypertension, diabetes, hyperlipidemia) and presence or absence of VAH in patients with PCI [19]. Peren et al. reported hyperlipidemia, diabetes, hypertension, and smoking were equally frequent in PCI or other location infarcts with or without VAH [12]. Hu et al. noted coronary artery disease association with lower risk of PCI by 2.2 times [14]. Hypertension, smoking, diabetes, hyperlipidemia, atrial fibrillation, coronary artery disease were the most common risk factors in study with 2245 patients with cerebral infarction conducted by Zeng et al. [20]. Two factors were found to be associated with PCI over ACI: male gender (OR = 1.39) and diabetes (OR = 1.67) and two factors were found to be associated with ACI over PCI: atrial fibrillation (OR = 1.89) and heart valve disease (OR = 2.31).

There were several limitations in this study. This was retrospective study conducted at single medical center. No data on VA blood flow velocity were collected. Ultrasound measurement is highly examiner dependent. Vascular risk factors were not collected for subjects in control group.

## 5. Conclusions

Vertebral artery hypoplasia is not a rare finding in individuals without symptoms of cerebrovascular accident, but more frequent in patients with vertebrobasilar cerebral infarction or vertebrobasilar artery syndrome. Vertebral artery hypoplasia can be considered a risk factor for posterior circulation infarction in subjects under 65 years of age.

## Figures and Tables

**Table 1:** Vertebral artery diameter and prevalence of vertebral artery hypoplasia in subjects with PCI/VAS, ACI and subjects without symptoms of cerebrovascular accident.

Variable	Subjects with PCI/VAS	Subjects with ACI	Control
Age, years	60.34 ± 12.77 ^a,b^	67.16 ± 12.89 ^a^	65.15 ± 12.90 ^b^
Right VA diameter, mm	2.96 ± 0.72 ^a,b^	3.17 ± 0.65 ^a^	3.16 ± 0.64 ^b^
Left VA diameter, mm	3.23 ± 0.70 ^a,b^	3.50 ± 0.68 ^a^	3.46 ± 0.65 ^b^
Non-dominant VA diameter, mm	2.68 ± 0.48 ^a,b^	2.94 ± 0.55 ^a^	2.91 ± 0.53 ^b^
Total VA diameter, mm	6.37 ± 0.82 ^a,b^	6.81 ± 0.89 ^a^	6.71 ± 0.81 ^b^
VA diameter < 3 mm, n (%)	99 (74.44) ^a,b^	44 (55.00) ^a^	296 (56.0) ^b^
VA diameter < 2.7 mm, n (%)	72 (54.14) ^a,b^	29 (36.25) ^a^	189 (35.7) ^b^
VA diameter < 2.5 mm, n (%)	45 (33.83) ^a,b^	12 (15.00) ^a^	104 (19.7) ^b^
VA diameter < 2.2 mm, n (%)	13 (9.77)	4 (5.00)	34 (6.4)

VA—vertebral artery; PCI—posterior circulation infarction; VAS—vertebrobasilar artery syndrome; ACI—anterior circulation infarction. ^a^—*p* < 0.05 comparing subjects with PCI/VAS and subjects with ACI. ^b^—*p* < 0.05 comparing subjects with PCI/VAS and control group subjects.

**Table 2:** Risk of PCI/VAS by degree of vertebral artery hypoplasia, OR (95% CI), *p*.

Variable	Subjects < 65 Years	Subjects 65+ Years
VA diameter ≥ 3 mm	Ref.	Ref.
VA diameter 2.7–2.9 mm	2.21 (1.02–4.80), *p* = 0.046	1.18 (0.51–2.76), *p* = 0.697
VA diameter 2.5–2.6 mm	2.36 (1.06–5.27), *p* = 0.036	1.91 (0.84–4.30), *p* = 0.121
VA diameter 2.2–2.4 mm	4.12 (1.95–8.70), *p* < 0.001	1.47 (0.54–3.98), *p* = 0.449
VA diameter < 2.2 mm	2.79 (1.06–7.29), *p* = 0.037	2.19 (0.65–7.36), *p* = 0.207

Ref.—reference; PCI—posterior circulation infarction; VAS—vertebrobasilar artery syndrome.

**Table 3:** The prevalence of vascular risk factors in subjects with posterior circulation infarction/vertebral artery syndrome and anterior circulation infarction.

Variable	Subjects with PCI/VAS	Subjects with ACI	*p*
Arterial hypertension	106 (79.7)	71 (88.8)	0.088
Diabetes	12 (9.0)	19 (23.8)	0.003
Ischemic heart disease	21 (15.8)	29 (36.2)	0.001
Atrial fibrillation	11 (8.3)	16 (20.0)	0.013

PCI—posterior circulation infarction; VAS—vertebral artery syndrome; ACI—anterior circulation infarction.

**Table 4:** Prevalence of vertebral artery hypoplasia among patients with cerebral infarction.

Author	Country	Definition of VAH	Number of Patients	VAH Prevalence, %	
				PCI	ACI
Hu et al. [14]	China	<2.0 mm	841	17.0	8.5
Chi et al. [13]	China	≤2.2 mm	353	44.8	22.4
Park et al. [11]	Korea	≤2.0 mm	529	45.6	27.1
Peren et al. [12]	Switzerland	≤2.5 mm	725	13	4.6
Kulyk et al. [4]	Italy	<2.5 mm	750	33.7	14.0
Sauer et al. [15]	Germany	≤2.5 mm	815	17.1	11.7
Mitsumura et al. [16]	Japan	<3.0 mm	129	44.4	24.7
Our study	Lithuania	<2.5 mm	213	33.8	15.0
Our study	Lithuania	<3.0 mm	213	74.4	55.0

## Data Availability

The data presented in this study are available on request from the corresponding author.
